# Correlates of Canadian mothers’ anger during the postpartum period: a cross-sectional survey

**DOI:** 10.1186/s12884-022-04479-4

**Published:** 2022-02-28

**Authors:** Christine HK Ou, Wendy A. Hall, Paddy Rodney, Robyn Stremler

**Affiliations:** 1 School of Nursing, University of Victoria, 3800 Finnerty Road, HSD Building, Room A402a, Victoria, British Columbia V8P 5C2 Canada; 2 Institute of Aging and Lifelong Health, University of Victoria, Victoria, Canada; 3 School of Nursing, University of British Columbia, 2211 Wesbrook Mall, Vancouver, BC V6T 2B5 Canada; 4 Lawrence S. Bloomberg Faculty of Nursing, University of Toronto, 155 College Street, Toronto, ON M5T 1P8 Canada

**Keywords:** Postpartum Anger, Postpartum Depression, Mothers’ Sleep, Infant Sleep, Mothers’ Cognitions about Sleep

## Abstract

**Background:**

Although some women experience anger as a mood problem after childbirth, postpartum anger has been neglected by researchers. Mothers’ and infants’ poor sleep quality during the postpartum period has been associated with mothers’ depressive symptoms; however, links between mothers’ sleep quality and postpartum anger are unclear. This study aimed to determine proportions of women with intense anger, depressive symptoms, and comorbid intense anger and depressive symptoms, and to examine mothers’ and infants’ sleep quality as correlates of postpartum anger.

**Methods:**

This cross-sectional survey study was advertised as an examination of mothers’ and babies’ sleep. Women, with healthy infants between 6 and 12 months of age, were recruited using community venues. The survey contained validated measures of sleep quality for mothers and infants, and fatigue, social support, anger, depressive symptoms, and cognitions about infant sleep.

**Results:**

278 women participated in the study. Thirty-one percent of women (*n* = 85) reported intense anger (≥ 90th percentile on State Anger Scale) while 26% (*n* = 73) of mothers indicated probable depression (>12 on Edinburgh Postnatal Depression Scale). Over half of the participants rated their sleep as poor (*n* = 144, 51.8%). Using robust regression analysis, income (*β* = -0.11, p < 0.05), parity (*β* = 0.2, p < 0.01), depressive symptoms (*β* = 0.22, *p* < 0.01), and mothers’ sleep quality (*β* = 0.10, *p* < 0.05), and anger about infant sleep (*β* = 0.25, *p* < 0.01) were significant predictors of mothers’ anger.

**Conclusions:**

Mothers’ sleep quality and anger about infant sleep are associated with their state anger. Clinicians can educate families about sleep pattern changes during the perinatal time frame and assess women’s mood and perceptions of their and their infants’ sleep quality in the first postpartum year. They can also offer evidence-based strategies for improving parent-infant sleep. Such health promotion initiatives could reduce mothers’ anger and support healthy sleep.

## Background

Perinatal mood and anxiety disorders (PMADs) are amongst the most common comorbidities of childbearing [1]. A global meta-analysis of postpartum mood disturbances drawing on 291 studies indicated a pooled prevalence of 17.7% [2] for depressive symptoms and 20.7% for anxiety [3] in the first postpartum year. In contrast, persistent and intense anger as a postpartum mood problem has been neglected; it is unclear how many women experience postpartum anger. The transition to parenthood can be stressful because women have to navigate physical changes and meet around-the-clock infant-care demands, while experiencing decrements to their sleep, personal autonomy, and time for self and others [4, 5]. For mothers, lack of sleep, fatigue, and carrying the majority of infant care responsibilities, mixed with the reduced capacity to meet their needs, can contribute to their experiences of anger [6].

Growing evidence suggests that persistent and intense anger is an important and overlooked PMAD [5–7]. In the postpartum time frame, there are significant costs to neglecting anger because evidence suggests that high levels of anger are associated with chronic and recurring symptoms of postpartum depression [7–9]. Depressive symptoms have been associated with poorer child socioemotional development [10] and worse partner mental health [11]. Anger in the context of postpartum depression has also been implicated in less positive mother-infant interactions. Field and colleagues [12] compared a sample of depressed mothers with high and low anger levels and found that mothers with high anger levels showed fewer positive behaviors towards infants (less smiling, gameplaying, and imitative behaviors). Moreover, the infants of mothers with depression and high anger levels displayed more negative affect (less smiling and vocalizing, and more distress behaviors). Despite emerging evidence that women can experience persistent and intense anger and even rage during the postnatal period [13], and that anger is associated with deleterious effects on maternal-child relationships, the proportion of women who experience intense anger after childbirth is not known.

Fatigue can compound the negative effects of anger on maternal-child relationships; mothers with moderate levels of fatigue identified higher levels of depressive symptoms and anxiety, parenting hostility, and lower levels of parenting warmth and involvement (Giallo et al., 2011). Women’s perceptions of poor sleep quality after childbirth have been associated with fatigue [14, 15]. Women’s postnatal sleep is characterized by fragmentation from waking to feed and tend to infants at night which in turn lowers sleep efficiency (the percentage of time spent in actual sleep) and reduces sleep duration [16–18]. In a meta-analysis of 28 studies, Yang et al. [19] found that the proportion of women with problematic sleep during the postpartum period was 67.2% (*95% CI* [57.6-75.5%]). A body of evidence suggests that perceptions of sleep quality mediate the relationship between actual sleep characteristics (e.g., latency, duration, efficiency) and depressive symptoms [16, 20, 21].

Most studies have examined women’s sleep outcomes in the first half of the postpartum year [19, 22]. The second half of the postpartum year, the time when infant sleep patterns are consolidating, has largely been neglected in research [22, 23]. Despite infant sleep problems being known to persist well into the first and even second and third postpartum years [24], research about ongoing parental sleep quality is sparse. There are robust associations between women’s poor sleep quality, fatigue, and postpartum depressive symptoms [11, 22, 25, 26]. Krizan and Hisler [27] found that adults’ sleep restriction has been associated with anger. Anger may be the result of increased sensitivity to negative stimuli from restricted sleep [28] and reduced capacity to regulate emotions [29].

It is important to note that PMADs also have psychosocial correlates, such as socioeconomic disadvantage [2, 30], poor social support, and stressful life events [30, 31]. Anger has been specifically linked with socioeconomic status; financial hardships have been associated with higher levels of anger [32, 33]. Inadequate social support could also be associated with anger because anger has been linked to unfulfilled expectations of others [6, 34]. For example, mothers may have expectations of receiving support from partners or family members and, when support does not materialize, women may feel distressed and angry [35].

Unfulfilled expectations of others fit with the concept of relational autonomy. Relational autonomy is a philosophical perspective that conceptualizes autonomy as a relational rather than individualistic practice [36]. Relational autonomy served as a theoretical framework for examining mothers’ anger and the causes of mothers’ anger during the postpartum period. Underpinning relational autonomy is the assumption that social relations, norms, and structures are simultaneously a precondition of, and an influence on, an individual’s ability to have and exercise autonomy [36–38]. Becoming a mother involves learning to manage one’s own needs and an infant’s needs, a process that is made easier with social and structural support [38]. Using the framework of relational autonomy points to indicators of socioeconomic status (i.e., income, immigration) and perceived availability of social support as variables that could contribute to explanations of women’s anger.

### Research aim & questions

Our study was designed to address the gaps in the investigation of relationships between mothers’ sleep quality, infant sleep quality, psychosocial factors, depressive symptoms, and mothers’ state anger. The purpose of this study was to examine the relationships between anger, depressive symptoms, and perceptions of mothers’ and infants’ sleep quality through the following research questions:

- **Research question 1:** What are the proportions of mothers reporting mood (intense anger or depressive symptoms) and poor sleep quality?
-
  - **Research question 1a:** What is the proportion of mothers reporting elevated depressive symptoms and intense anger?
  - **Research question 1b:** What is the proportion of mothers reporting poor sleep quality and intense anger?
- **Research question 2:** What are the bivariate relationships between depressive symptoms, mothers’ and infants’ sleep quality, fatigue, social support, demographic characteristics and mothers’ anger?
- **Research question 3:** What variables significantly predict mothers’ anger?

## Methods

The data presented here are from the quantitative portion of a mixed methods study about mothers’ anger in the latter half of the first postpartum year. A cross-sectional survey study of Canadian mothers was undertaken after receiving ethical approval from our university-based behavioral research ethics board (H18-03761). All study procedures were performed in accordance with university and Canadian Tri-Council Policy Statement guidelines. All participants completed the informed consent process. Biological mothers of healthy singleton infants between six and twelve months of age living in Canada were invited to participate in the survey study. Women completing a screening survey were ineligible if they had a history of diagnosed depression (during pregnancy or prior to pregnancy), had a chronic health or developmental condition that affected their sleep, were prescribed medication for sleep, were employed working night shifts, or had a baby that was delivered prematurely. Eligible participants were recruited via social media (Facebook and Twitter), internet community boards, and study posters at community centers. Women who viewed the study advertisement (online and in-person) and who were interested in participating were provided with a link to the screening survey to determine eligibility. Those who were eligible were emailed a link to the survey study. Data were collected between April and July of 2019. A list of Canadian resources to support mental health was made available for all participants at the end of the survey. This was particularly important for participants who may have been distressed following the survey due to the sensitive nature of the questions about anger and depressive symptoms.

### Measures

The survey contained measures of sleep quality, mood (depression and anger), social support, and demographic variables. Demographic variables examined included mothers’ age, relationship status, number of children at home, employment status, immigration status, level of education obtained, household income, and infant age and sex. The study variables relating to mood, sleep, and support were operationalized with the following measures:

The State Anger Scale (SAS), a subscale from the State-Trait Anger-Expression Inventory-2 (STAXI-2), was used to measure mothers’ state anger levels. State anger reflects a psychobiological and transient condition marked by subjective feelings that vary in intensity (e.g., annoyance, rage) whereas trait anger reflects individual temperamental disposition to anger [39]. We were interested in capturing mothers’ experiences of state anger, in response to their circumstances. The SAS assessed current feelings of anger with items such as “I am furious”, and “I feel like yelling at somebody”. The SAS has 15 items with each item being rated on a four-point Likert-like scale with a scoring range between 15 and 60. A score of 27 (equivalent to the 90^th^ percentile on norms established for non-psychiatric women by Spielberger, 1999 [40]) was used as the cut-off for indicating intense anger levels. Studies that have used the SAS from the STAXI-2 for non-clinical samples of women have indicated good internal consistency with Cronbach’s α of 0.75 to 0.93 [41, 42]. For this study, the Cronbach’s alpha for the SAS was 0.92, indicating excellent internal consistency. Anger levels were dichotomized as intense and less intense based on normed percentiles (with 90^th^ percentile and above indicating intense anger levels) on the SAS for non-psychiatric women (Spielberger, 1999) in order to answer the first research question about the proportion of women experiencing intense anger. The SAS total score was used as the dependent variable in the regression analysis of the predictors of mothers’ anger.

The Edinburgh Postnatal Depression Scale (EPDS), developed for the purpose of detecting possible postpartum depression in women [43], contains ten self-report questions that measure the presence of depressive and anxiety symptoms in the previous 7 days. The scale is amongst the most established and frequently used measures for detecting PMADs [44, 45]. Scores can range between 0 and 30. Cox et al. (1987) [43] recommended a cut-off of >12 for detecting probable depression. A psychometric evaluation of the measure demonstrated that internal reliability was adequate with Cronbach’s α ranging from 0.73 to 0.78 [44]. For this study, the Cronbach’s alpha was 0.84, indicating good internal consistency. The presence of probable depression was determined using the EPDS cut-off score of >12 in order to answer the first research question. The total EPDS score was used as an independent variable predicting mothers’ anger in the regression analysis.

The Pittsburgh Sleep Quality Index **(**PSQI) is a generic measure of sleep quality that can discriminate between ‘good’ and ‘poor’ adult sleepers [46] and is comprised of 19 items divided into seven components (sleep duration, latency, quality, efficiency, disturbance, use of sleep medications, and daytime dysfunction) that provide a summary of self-reported sleep characteristics. The PSQI has been used widely to determine women’s sleep quality during the perinatal period [19, 47]. Because we were examining how mothers’ perceptions of sleep quality were associated with anger, we used the perceived sleep quality (SQ) component of the PSQI, which consists of a single-item asking “During the past month, how would you rate your sleep quality overall?”. In order to answer the first research question, we dichotomized sleep as good if participants selected “fairly good” or “very good” and poor if they selected “fairly bad” or “very bad”. The SQ component of the PSQI and other single-item measures have been used to measure parental perceptions of sleep quality [48–51]. A sleep quality measure derived from the PSQI single item has been psychometrically tested against the full PSQI scale with adequate convergent validity (*r =* -.72, 95% CI [-0.68, -0.76]) [52]. Two-day interval test-retest reliability of the PSQI SQ item was adequate with *r =* .76 [52].

The Brief Infant Sleep Questionnaire (BISQ), developed as a screening tool for infant sleep problems [53], was used to assess infant sleep characteristics and parental perceptions about infant sleep. The measure has been validated for web surveys across cultures [54]. Sadeh (2004) [53] demonstrated that there were significant differences in the infant sleep characteristics (i.e., sleep latency, day and nighttime sleep duration, number of night wakes, and time spent awake at night), which would be expected to be associated with problematic infant sleep, and parental rating of infant sleep problem severity (“no problem”, “small-to-moderate problem”, and “serious problem”). Thus for this study, infant sleep quality was assessed using a single item regarding parents’ perceptions of the presence and degree of infant sleep problems.

The Multidimensional Assessment of Fatigue (MAF) is a measure that we used to assess mothers’ fatigue. The MAF comprises 16 items assessing fatigue severity, distress about fatigue, and interference with activities of daily living. For this study, a global fatigue index was calculated from items and ranges from 1 (no fatigue) to 50 (severe fatigue) and used as a continuous variable to predict mothers’ anger. The MAF has been validated for use with women during the perinatal period. Cronbach’s alpha has ranged from 0.94 to 0.95 [55, 56]. For this study, the Cronbach’s alpha was 0.93, indicating excellent internal consistency.

The Maternal Cognitions about Infant Sleep Questionnaire (MCISQ) is a 20-item survey that assesses the presence of aversive parental thoughts about managing infant sleep; it is comprised of 5 subscales including limit setting around sleep, anger about infant sleep, doubts about sleep management, nighttime feeding necessity, and infant safety [57]. Higher scores indicate a greater degree of difficulty in managing infant sleep. The anger about infant sleep subscale was of particular interest as a potential correlate with mothers’ state anger. Morrell [57] found the measure’s overall internal consistency was 0.82. Cronbach’s alpha for the whole MCISQ was 0.82 and 0.59 for the 5-item anger subscale. For this study, the anger about infant sleep subscale was the predominant variable of interest so it was used as a predictor variable for mothers’ state anger.

The Family Support Scale (FSS) is an 18-item scale used to examine the availability and frequency of support (e.g., from partners, kin, health care providers, and community agencies) for parents of young children [58]. Each available source of support is ranked on a Likert scale between 1 (not at all helpful) and 5 (extremely helpful). Although originally developed for parents of children with special needs, the FSS has been used in the context of perinatal depression [59] and parental stress after childbirth [60], with Cronbach’s alpha being 0.70, and 0.72 for each study respectively. In this study, Cronbach’s alpha was 0.69, indicating poor to fair internal consistency. For the purposes of this study, the FSS total score reflected the degree of social support received and was used as a continuous variable to predict mothers’ anger.

### Data analysis

Data analyses were performed on 278 surveys using R (RStudio, version 1.2.5033), an open-source statistical program. After checks for data integrity and data screening and cleaning, univariate and bivariate descriptive statistics for participants’ demographic, sleep, and mood characteristics were calculated. Bivariate relationships between variables of interest were examined using a correlation table and a series of chi-square and t-tests to identify significant associations. Correlations between characteristics were examined and variables that were moderately or highly correlated with anger were used for multivariate regression model building with state anger as the outcome variable. A priori variables that were tested included income, education, immigration status, depressive symptoms, fatigue, mothers’ sleep quality, angry cognitions about infant sleep, and social support. Parity was included because women’s report of the number of children demonstrated a significant correlation with anger. Examination of the residuals in the linear models revealed moderate violations of the assumptions of equal variances (heteroscedasticity) and normality. While moderate degrees of non-normality can generally be tolerated in linear regression, heteroscedasticity can lead to biased standard errors and significance values [61, 62]. Robust regression technique (using robustbase package version .93-6) was used to correct for heteroscedasticity [61, 63] and to improve the accuracy of beta coefficient and significance estimates in the final model [61, 64]. Robust models do not produce conventional goodness of fit tests like the F-test [65].

## Results

Six hundred and twelve women received an invitation to the survey after completing an online screening survey, with 331 participants starting the actual survey. Forty-four participants dropped out of the survey; their responses were not retained for this analysis because those respondents did not complete the anger measure (the outcome variable of interest). A further 9 cases were excluded because of evidence of participant ineligibility (e.g., having an infant with health problems or premature delivery). Participants who were excluded because they withdrew or were ineligible did not differ significantly from other participants with respect to maternal age, infant age, parity, education, or income; however, excluded participants tended to be employed, and this bordered on significance (χ^2^ (1, [*N* = 326]) *=* 3.719, *p =* 0.054).

### Sample characteristics

The final sample consisted of 278 women between the ages of 23 and 44 years (*M* = 32.6, *SD* = 3.77) living across Canada, with the majority (77%, *n* = 220) being 30 years of age and over (Table 1). The sample reflected  high socioeconomic status with 73.0% (*n* = 203) holding a university degree or higher and 69.8% (*n* = 194) having household incomes of ≥ $90,000 CAD. All but 3 participants were partnered and just over half (53.6%, *n* = 149) were first-time mothers.

**Table 1:** Sample characteristics

Characteristic		All Participants *N* = 278 Mean, SD / n (%)	Anger Level^**a**^		*P*-value
			Less Intense *N* = 193; n (%)	Intense *N* = 85; n (%)	
Age	Range 23-44 years	32.6 ± 3.8	32.6 ± 3.7	32.7 ± 4.1	0.97
Infant age	Range 6-12 months	8.6 ± 1.9	8.5 ± 2.0	8.7 ± 1.9	0.43
Infant sex	Male	148 (53.4)	105 (54.4)	43 (50.6)	0.72
Partnered	Yes	275 (98.9)	192 (99.5)	83 (97.6)	0.46
Parity	Primiparous	149 (53.6)	1.5 (0.73)	1.8 (0.76)	**<0.01**
	Multiparous	128 (45.0)			
	Unknown	1 (<1)			
Employment	Yes	50 (18.0)	31 (16.1)	19 (22.4)	0.28
Immigrant	Yes	41 (14.7)	33 (17.1)	8 (9.5)	0.14
Education	Postgraduate	81 (29.1)	64 (33.2)	17 (20.0)	**<0.01**
	University	122 (43.9)	84 (43.5)	38 (44.7)	
	College	62 (22.3)	41 (21.2)	21 (24.7)	
	High School	13 (4.7)	4 (2.1)	9 (10.6)	
Household income	>$110K	134 (48.2)	104 (53.9)	30 (35.3)	0.01
	$90,000-109,999	60 (21.7)	37 (19.2)	23 (27.1)	
	$60,000-89,999	60 (21.7)	40 (20.7)	20 (23.5)	
	<$60,000	23 (8.3)	11 (5.7)	12 (14.1)	
	Unknown	1 (<1)			
Anger (SAS)	Range 15-53	24 ± 7.6	20.0 ± 3.5	33 ± 6.3	**< 0.01**
Depressive symptoms (EPDS)	Range 0-21	9.3 ± 4.7	8.2 ± 4.4	11.8 ± 4.3	**< 0.01**
EPDS score >12	Yes	73 (26.0)	34 (46.6)	39 (53.4)	**< 0.01**
Sleep Quality (SQ)	Range 1-4	2.6 ± 0.7	2.4 ± 0.7	2.8 ± 0.6	**< 0.01**
Poor sleep	Yes	144 (52.0)	85 (44.0)	59 (69.4)	**< 0.01**
Fatigue (MAF)	Range 7-45	30.3 ± 8.5	28.5 ± 8.7	34 ± 6.4	**< 0.01**
Infant sleep problem (BISQ)	Yes	194 (69.8)	122 (62.3)	72 (84.7)	**< 0.01**
Maternal cognitions about infant sleep (MCISQ total)	Range 5-69	34.9 ± 12.7	33 ± 12.7	39 ± 2.0	**< 0.01**
Anger (subscale)		6.4 ± 3.9	5.5 ± 3.5	8.5 ± 4.0	**< 0.01**
Limit Setting (subscale)		14.9 ± 6.0	14.7 ± 6.1	15.5 ± 5.6	0.26
Doubts (subscale)		7.0 ± 4.5	6.5 ± 4.3	8.1 ± 4.6	**< 0.01**
Safety (subscale)		3.2 ± 2.4	2.9 ± 2.4	3.6 ± 2.4	0.07
Feeding (subscale)		6.5 ± 3.7	6.3 ± 3.8	7.0 ± 3.3	0.12
Family support total score (FSS)	44.8 (9.4)	44.8 ± 9.4	44.7 ± 9.4	45.0 ± 9.4	0.82

^a^State Anger Scale raw score of 27 (90^th^ percentile). *Note:* *BISQ* Brief Infant Sleep Questionnaire, *EPDS* Edinburgh Postnatal Depression Scale, *FSS* Family Support Scale, *MAF* Multidimensional Fatigue Scale, *MCISQ* Maternal Cognitions about Infant Sleep, *SAS* State Anger Scale, *SQ* Sleep Quality

### Research Questions 1, 1a, 1b

To answer RQ1 about the proportions of women who experienced intense anger, elevated depressive symptoms, and poor sleep quality, 30.6% of women (*n* *=* 85) reported intense anger, 26% (*n* = 73 ) indicated elevated depressive symptoms, and 51.8% (*n* = 144) reported poor sleep quality. To answer RQ1a about the proportion of women who experienced elevated depressive symptoms and intense anger, 14% (*n* = 39) experienced elevated depressive symptoms and intense anger (Table 2). A chi-square test demonstrated that women who had depressive symptoms were nearly 4 times more likely to experience intense anger relative to those who did not have depressive symptoms above cut-off (OR = 3.96, 95% CI [2.25, 6.98], *p* < 0.001). Of the 85 women who had intense anger, almost half (*n* = 39) experienced probable depression; however, it is noteworthy that a large proportion of women (*n* = 46) had intense anger but did not have depressive symptoms above cut-off. To answer RQ1b about the proportion of women who experienced poor sleep quality and intense anger, 21% of women (*n =* 59) experienced poor sleep quality and intense anger (Table 3). Women who perceived their sleep as poor (fairly bad or very bad), were 2.9 times more likely to have intense anger compared to mothers who perceived their sleep as good (fairly good or very good) (OR = 2.88, 95% CI [1.68, 4.96], p < 0.001).

**Table 2:** Proportions of intense anger and probable depression

Anger	Probable Depression		
	**Yes**	**No**	**Total**
**Intense Anger**	39 (14.0%)	46 (16.5%)	85 (30.6%)
**Less Intense Anger**	34 (12.2%)	159 (57.2%)	193 (69.4%)
**Total**	73 (26.3%)	205 (73.7%)	278 (100%)

***Χ***^***2***^* = 22.9 ****df**** = 1 ****p**** < .001*

***O.R. ****(95% C.I.) = ****3.96**** (2.25 – 6.98)*

**Table 3:** Proportions of intense anger and mothers’ sleep quality

Sleep Quality	Anger		
	**Intense**	**Less Intense**	**Total**
Poor	59 (21.2%)	85 (30.6%)	144 (51.8%)
Good	26 (9.4%)	108 (38.8%)	134 (48.2%)
**Total**	85 (30.6%)	193 (69.4%)	278 (100%)

***Χ***^***2***^* = 14.2135 ****df**** = 1 ****p**** = .0002*

***O.R. ****(95% C.I.) = ****2.88**** (1.68 - 4.96)*

### Research Question 2

Table 4 delineates the bivariate correlations between anger scores and demographic variables, both mothers’ and infants’ sleep quality, and mothers’ perceptions of fatigue, depressive symptoms, angry cognitions about infant sleep, and social support

**Table 4:** Pearson and spearman correlations of anger and particular variables

Variable	1	2	3	4	5	7	8	9	10
1. Anger	1.000								
2. Parity	0.17**	1.000							
3. Education	-0.20**	-0.17**	1.000						
4. Income	-0.18**	0.03	0.23**	1.000					
5. Depressive symptoms	0.42**	-0.04	-0.20**	-0.21**	1.000				
7. Mothers’ sleep quality	0.32**	0.10	-0.13*	-0.15*	0.27**	1.000			
8. Fatigue	0.38**	0.04	-0.04	-0.21**	0.50**	0.54**	1.000		
9. MCISQ anger	0.42**	-0.03	-0.09	-0.14*	0.32**	0.32**	0.42**	1.000	
10. Support	-0.02	0.08	0.14*	0.06	-0.11	-0.05	-0.10	-0.06	1.000

*Note.* * indicates *p* < 0.05. ** indicates *p* < 0.01. *MCISQ* Maternal Cognitions about Infant Sleep

Participants who had intense anger (*n* = 85, M = 33 ± 6.3) had more children, t(152.8) = 2.8, *p* < 0.01, and higher scores on depressive symptoms, t(160.1) = 6.3, *p* < 0.01, fatigue t(212.2) = 6.01, *p* < 0.01, perceptions of the severity of infant sleep problems, χ^2^ (1, *n* = 278) = 11.9, *p* < 0.01, dysfunctional cognitions about infant sleep, t(168.1) = 3.76, *p* < 0.01, and anger about infant sleep t(144.1) = 6.03, *p* < 0.01 (Table 1). They also reported lower education levels, χ^2^ (3, *n* = 278) = 12.9, *p* < 0.01, income, χ^2^ (3, *n* = 277) = 11.18, *p* = 0.01, and perceived sleep quality, χ^2^ (1, *n* = 278) = 14.2, *p* < 0.01 (Table 1).

### Research Question 3

Preliminary model building was carried out using robust regression (Table 5) to answer the third question about variables associated with mothers’ anger. In the first step, demographic variables explained a small proportion of the variance in anger, with income and parity demonstrating significant associations, R^2^ = 0.124. In the second step, depressive symptoms, sleep quality, fatigue, anger about infant sleep, and social support contributed to a model that explained 38% of the variance in mothers’ anger, R^2^ = 0.376; parity, depressive symptoms, and anger about infant sleep were significantly associated with mothers’ anger. Given that large standard errors were identified for fatigue and sleep quality and that these constructs have theoretical overlap, in the third step of model development, when fatigue was removed, sleep quality was significantly associated with mothers’ anger (with worse perceived sleep quality associated with greater anger), R^2^ = 0.371. In the final robust regression model, income was associated with mothers’ anger (*β* = -0.11, *p <* 0.05). Education (*β* = -0.05, *p* = 0.29) and immigration status (*β* = -0.03, *p* = 0.43) were not significantly associated with mothers’ anger. Mothers’ sleep quality (*β* = 0.10, *p =* 0.02) and anger about infant sleep (*β* = 0.25, *p* < 0.01) were associated with mothers’ anger. Contrary to expectations, social support was not associated with anger in our model (*β* = 0.07, *p* = 0.11).

**Table 5:**
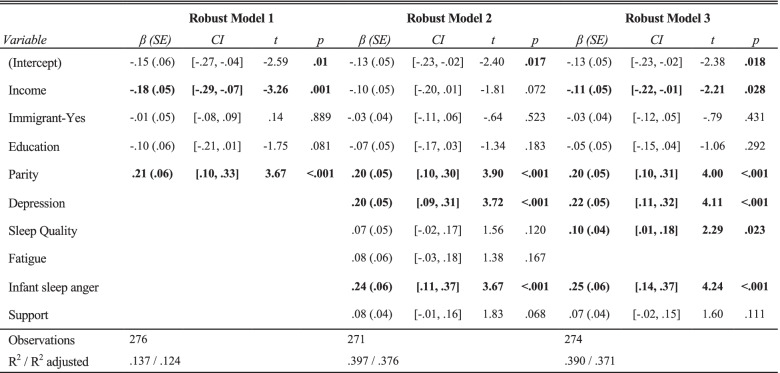
Robust regression model building explaining mothers’ anger

*Note:* Beta coefficients are standardized. **Bolding** indicates statistical significance

## Discussion

Almost one third of women in this study experienced intense anger and more than a quarter experienced probable depression. Of the women who experienced intense anger, about 50% experienced concurrent probable depression while the other 50% did not. Mothers’ anger has typically been explored in the context of depression, but our finding suggests that anger is a distinct mood disturbance that is not consistently associated with depressive symptoms.

There is remarkable heterogeneity in the presentation of ‘postpartum depression’, an umbrella term that covers a constellation of symptoms which may include depressive symptoms, anxiety, irritability, hypervigilance, and intrusive thoughts, in addition to anger [66]. A transdiagnostic approach to understanding perinatal depression is gaining momentum as an alternate framework to understand mental illness that bypasses the traditional DSM-5 diagnostic boundaries of mental health problems; it recognizes that psychological distress manifests in many ways, with symptoms occurring on a continuum [67, 68]. As such, when assessing PMADs, we ought to consider anger, depressive symptoms, and anxiety, among other conditions, and place them in a multidimensional view of perinatal psychological distress.

The finding that intense anger can occur, in the absence of mothers’ report of probable depression suggests anger requires attention as a distinct dimension of postpartum psychopathology. Identifying anger as a distinctive perinatal mood problem suggests that clinicians and researchers ought to be screening for anger when investigating PMADs. More work is needed to explore the possible origins of perinatal anger (e.g., trait anger, antenatal anger, adverse childhood experiences, intimate partner violence), its general effects, and its consequences for maternal-infant dyadic attachment, maternal functioning and efficacy, and parental relationships. Anger can be understood as a highly social emotion [33, 69, 70], which is more often than not fueled by social context and situations involving others. The study findings support the social nature of anger. A recent systematic review of partners’ experiences indicated that partners experience distress, anger, relationship conflict and even breakdown in the context of women’s PMADs [69].

The framework of relational autonomy suggests that affective responses (e.g., anger and sadness) are reactions to constrained personal autonomy. Lower income was associated with higher anger levels. Inadequate income has been linked with feelings of powerlessness and hardship, which are associated with anger in women [6, 71]. It is possible that governmental policies that provide financial support through child and family benefits and provisions for childcare, and that sponsoring parenting programs to enhance parenting confidence can help women experiencing economic hardship to reduce their anger. Comparisons of parental-child outcomes between groups (e.g., in different municipal areas, states, or provinces) who have received financial assistance and/or parenting support and those who have not could help to demonstrate the need for and efficacy of systemic supports, controlling for socioeconomic and family related variables.

It is also important to consider that predisposition to anger, as a personality characteristic, influences the experience of state anger [39]. Higher trait anger may explain why some women in this study experienced intense anger in the absence of elevated depressive symptoms or poor sleep quality. Tobe and colleagues [72] identified a relationship between trait anger and postnatal depression; however, they did not examine state anger. Future studies should investigate the impact of trait anger on parenthood and mothers’ mental health longitudinally.

Contrary to our expectations, social support had little effect on mothers’ anger in this study. We suspect that the FSS measure did not adequately capture the types of supports that were important for participants, such as the provision of instrumental or emotional support. We also suggest that partner support was not adequately weighted in this measure. In romantic relationships and especially during childbearing, women expect their partners to be their greatest source of support [73, 74]. Lack of desired forms of support from partners was a significant cause of distress for women in other studies [75, 76]. A post-hoc analysis of partner support (a single item from the FSS) identified a very small and non-significant relationship between partner support and mothers’ anger (*β* = -0.01, *p =* .73). The use of a measure that contextualizes support for the postnatal period such as the Postpartum Partner Support Scale [75] may better capture the sufficiency of partner support for women.

Our finding that higher parity was associated with higher anger scores suggests that women struggle as they have more children; this result is contrary to findings that indicate that multiparous mothers do not require additional supports because they are experienced and have greater parenting efficacy [77, 78]. Our finding triangulates with previous findings that indicate an association between greater parity and elevated depressive symptoms [79, 80]. We argue that increased anger and depressive symptoms highlight the need for increased social and structural supports for women who have a second or third child, in response to differing infant temperaments, increased demands for mothers related to managing their own and children’s sleep and other needs, or to income insufficiency. In this study, it is noteworthy that one in four women experienced elevated depressive symptoms, higher than the one in six women indicated by Hahn-Holbrook and colleagues [2]. Similarly, a recent Statistics Canada survey reported that 23% of women endorsed feelings consistent with postpartum depression or anxiety [81]. Anthropologist Edward Hagen [82] argued that postpartum depression is an evolutionary adaptation that assists mothers to acquire adequate support (e.g., from partners and kin) to meet their physical (e.g., sleep) and emotional needs when they do not have enough external resources to care for themselves and their infant. It is possible that women resort to anger when expressing distress, sadness, or anxiety fails to elicit sufficient support from partners and kin or that some women have been socialized to display anger rather than sadness when experiencing distress [35].

To our knowledge, this is the first study that has examined the relationships between mothers’ state anger and perceived sleep quality after childbirth. Just over half of mothers reported poor sleep quality and mothers’ perceptions of poor sleep quality were associated with anger. Mothers’ state anger scores do not reveal what women are angry about; however, maternal anger about infant sleep provides a clear link between mothers’ anger and infant sleep problems. Maternal anger about infant sleep was also correlated with the perception of an infant sleep problem (r = 0.45, *p* < 0.01). Hall and colleagues reported that maternal anger about infant sleep and depressive symptoms could be reduced by managing infant sleep problems [56, 83]. Taken together, the study findings suggest that 1) it is critical to screen mothers and infants for sleep problems because they can be associated with mood problems [11, 84] and that 2) clinicians ought to provide education about infant sleep patterns and teach psycho-behavioral interventions to parents to promote maternal-infant sleep and manage sleep problems. Talking to parents about parental and infant sleep is a low-barrier and stigma free way for clinicians to initiate conversations about daily functioning and mental health [85]. Addressing maternal and infant sleep problems can contribute to managing postpartum mood problems given the empirical support for psycho-behavioral education around sleep contributing to improved maternal mental health [83, 86]. Education about parent-infant sleep and mood could serve as an important form of anticipatory guidance to prevent PMADs during pregnancy and after childbirth. However, many clinicians lack sleep related training and education, suggesting that more emphasis on assessment of sleep and managing sleep problems in disciplinary curriculums and continuing education is required [87–90].

Strengths of the study include a large, pan-Canadian sample. Framing this study as a study of mothers’ and infants’ sleep quality may have also helped to attract participants because inadequate sleep is a common postnatal challenge. We also used a high cut-off for depression (>12 on EPDS) and anger (≥90th percentile on SAS) such that those cut-off scores were more likely to capture women with clinically relevant levels of depressive symptoms and anger.

### Limitations and future research directions

This study has limitations that warrant caution with the interpretation of results. First, a convenience sampling frame was used, with the resulting sample being highly educated, financially secure, with lower levels of ethnic diversity. Second, this study was cross-sectional and only examined anger at one time-point; as such, it was not possible to know whether the anger captured was episodic or chronic in nature. Chronic experiences of anger would be of greater concern from an individual and family health perspective. A future longitudinal study of anger could differentiate between episodic and chronic anger and associated outcomes for women and the effects of anger on maternal-infant and family outcomes. Third, because the study was framed around mothers’ and infants’ sleep, the number of women with mood disturbances may be overrepresented relative to the general population because participants experiencing sleep problems and infant sleep problems may have been more likely to complete the survey. Only 30% of our sample indicated an absence of infant sleep problems as compared to 75-88% in other samples [91, 92] . Fourth, we did not employ objective measures for assessing sleep problems (e.g., actigraphy) or use a clinical interview for establishing the presence of depressive disorders for participants. Finally, contrary to our expectations and empirical support for the role of social support in reducing mothers’ anger, we were unable to demonstrate this relationship empirically. This may have been because the support measure that we used did not explicitly measure partner and family support in terms of maternal emotional adjustment. In future studies of anger, it would be important to examine the role of partner support specifically for mothers’ mental wellbeing, given that women most often expect partners to be a chief source of support.

## Conclusion

Research about mothers’ anger after childbirth and its connection to mothers’ and infants’ sleep remains in its infancy. This study extends our understanding of intense anger as a distinct PMAD. In agreement with the extant literature, mothers’ and infants’ sleep problems were associated with maternal mood disturbances in this study. Two distinct study findings emerged:, 1) anger does not consistently occur in the context of postpartum depressive symptoms; and 2) intense postpartum anger is associated with mothers’ perceptions of lower sleep quality for themselves and their infants. Anger was slightly more prevalent than depressive symptoms, suggesting that mothers’ anger in the postpartum period requires close attention by clinicians and researchers. Having more than one child, maternal anger about infant sleep, mothers’ perceptions of lower sleep quality, and higher levels of depressive symptoms were associated with mothers’ anger. Taken together, it is important to assess for the presence of intense anger and sleep problems in the first postpartum year and to educate health care providers, women, and families about the associations between poor quality sleep and postpartum emotional distress.

## Data Availability

Data can be made available from the corresponding author on request for non-commercial purposes.

## Abbreviations

BISQ: Brief Infant Sleep Questionnaire
EPDS: Edinburgh Postnatal Depression Scale
FSS: Family Support Scale
MAF: Multidimensional Assessment of Fatigue
PMAD: Perinatal Mood and Anxiety Disorders
PND: Postnatal Depression
PSQI: Pittsburgh Sleep Quality Index
SAS: State Anger Scale
